# SIGRN: Inferring Gene Regulatory Network with Soft Introspective Variational Autoencoders

**DOI:** 10.3390/ijms252312741

**Published:** 2024-11-27

**Authors:** Rongyuan Li, Jingli Wu, Gaoshi Li, Jiafei Liu, Jinlu Liu, Junbo Xuan, Zheng Deng

**Affiliations:** 1 Key Lab of Education Blockchain and Intelligent Technology, Ministry of Education, Guangxi Normal University, Guilin 541004, China; lryuan2@163.com (R.L.); ligaoshi@gxnu.edu.cn (G.L.); liujiafei@gxnu.edu.cn (J.L.); jlliu@mailbox.gxnu.edu.cn (J.L.); xuanjunbo@mailbox.gxnu.edu.cn (J.X.); zhengdeng1024@163.com (Z.D.); 2 Guangxi Key Lab of Multi-Source Information Mining & Security, Guangxi Normal University, Guilin 541004, China; 3 School of Computer Science and Engineering, Guangxi Normal University, Guilin 541004, China

**Keywords:** variational autoencoder, structural equation model, gene regulatory network, soft introspective adversarial

## Abstract

Gene regulatory networks (GRNs) exhibit the complex regulatory relationships among genes, which are essential for understanding developmental biology and uncovering the fundamental aspects of various biological phenomena. It is an effective and economical way to infer GRNs from single-cell RNA sequencing (scRNA-seq) with computational methods. Recent researches have been done on the problem by using variational autoencoder (VAE) and structural equation model (SEM). Due to the shortcoming of VAE generating poor-quality data, in this paper, a soft introspective adversarial gene regulatory network unsupervised inference model, called SIGRN, is proposed by introducing adversarial mechanism in building a variational autoencoder model. SIGRN applies “soft” introspective adversarial mode to avoid training additional neural networks and adding additional training parameters. It demonstrates superior inference accuracy across most benchmark datasets when compared to nine leading-edge methods. In addition, method SIGRN also achieves better performance on representing cells and generating scRNA-seq data in most datasets. All of which have been verified via substantial experiments. The SIGRN method shows promise for generating scRNA-seq data and inferring GRNs.

## 1. Introduction

Gene regulatory networks (GRNs), encoding the sophisticated regulatory interactions between transcription factors (TFs) and their target genes, have attracted substantial attention in the fields of medical and biological technologies. Understanding the interactions is essential for recognizing the underlying causes of diseases and can contribute to the progress of new therapeutic strategies [1]. Nevertheless, it is extremely laborious and costly to acquire them through biological experiments [2]. Fortunately, massive amounts of single-cell RNA sequencing (scRNA-seq) data have been available with the rapid progress of single-cell sequencing technologies. The study of inferring GRNs from scRNA-seq data via computational approaches has therefore become a reality and drawn considerable attention in recent years [3].

There are mainly two categories of approaches to infer GRNs. The first involves traditional non-deep learning techniques, such as mutual information [4,5], tree-based models [6,7], and linear models [8,9]. In these methods, in order to alleviate data sparsity issue of scRNA-seq data resulting from dropout events [10], an amount of genes and cells are filtered out during the preprocess. This may hinder the exploration of new regulatory networks. The second is based on deep neural networks (DNNs), which possess strong representation learning capabilities to effectively address data sparsity issues [11,12]. This study focuses on the latter, which is further categorized into supervised method and unsupervised one.

Several supervised computational frameworks have utilized convolutional neural networks (CNNs) to infer relationships from images encoded by pairs of genes [13,14,15]. In 2023, Xu et al. [16] regarded that the conversion of gene expression data into images has altered data format and may lose global information [17]. Then they put forward an interpretable transformer-based method STGRNS, which captures global connections through a gene expression motif technique. Since it is quite labor-intensive and expensive to identify transcription-factor targets [18], various unsupervised approaches were also devised. In 2021, Shu et al. [19] introduced method DeepSEM, which specifically represents the structure of GRNs by using two neural network layers. In 2023, Zhang et al. [20] presented method MetaSEM, a meta-learning framework designed to inference GRNs. That same year, Zhu et al. [21] indicated DeepSEM unstable results may produce inferred networks with great variations in performance, and put forward an extension of it called GRN-VAE. It enhances stability as well as robustness by introducing delay and dropout mechanisms. Nevertheless, due to the adoption of an additional classifier for conducting Dropout Augmentation, the number of model parameters may be increased.

In the above mentioned unsupervised methods, the correlations between observed and hidden variables are firstly extracted by using structural equation model (SEM), and the probability distribution of latent variables are learned with a variational autoencoder (VAE). All of them make full use of the potential of VAE in capturing complex structure in the data and generating realistic and diverse data samples. Nevertheless, it is usual for VAE to reconstruct low-quality data [22,23], which may negatively affect GRN inference. Generative Adversarial Network, an unsupervised approach based on game theory, possesses the outstanding capability of generating data. Recently, we proposed method IntroGRN [24] by introducing adversarial mechanism in building a VAE model. Although method IntroGRN performs well competitive to methods DeepSEM and GRN-VAE, its inference performance is very sensitive to two hyperparameters (parameters *m* and α) taken in its loss function. Extensive pre-experiments need to be done to ascertain them. Therefore, in this study, we put forward method SIGRN, which is an improved version of method IntroGRN by eliminating the above two parameters with a soft threshold mechanism.

In the SIGRN method, an introspective adversarial network (IAN) is introduced by respectively adopting the decoder and encoder of VAE as generator and discriminator, avoiding training additional neural networks. A new loss function is constructed to train the VAE and the IAN simultaneously, i.e., it focuses not only on the discrepancy between real data and generated one, the latent variables encoded by real data and those sampled from the prior distribution, but also on the discrepancy between the latent variables encoded by generated data and those sampled from the prior distribution. Although method SIGRN has a longer running time than method GRN-VAE due to implementing adversarial training, it outperforms several cutting-edge approaches across most benchmark datasets. Moreover, compared to the “hard” threshold mechanism in the IntroGRN method [24], the “soft” one in method SIGRN is free of being restricted by additional hyperparameters and overcomes gradient disappearance effectively, making the model training more stable.

## 2. Experimental Results and Analysis

In the section, extensive experiments were conducted using real biological datasets. The SIGRN method was implemented on the deep learning framework PyTorch. More detailed instructions for implementing it can be found at https://github.com/lryup/SIGRN, accessed on 1 July 2024. All experiments were performed in an Ubuntu 20.04 environment using NVIDIA A100 GPU with 40 GB of memory.

### 2.1. Data Preparation

To validate the performance of inferring GRN, eight scRNA-seq datasets were utilized in the experiments. Seven small-scale labeled datasets were acquired from the BEELINE benchmarks [25]. Additionally, a large-scale unlabeled dataset (Saline) was acquired from a published adult mice dataset [26]. As performed by Shu et al. [19] and Zhu et al. [21], 14 subdatasets were firstly constructed by providing two gene scales, i.e., “TFs+500” and “TFs+1000” (shown in Table 1) for each of the seven datasets. That is to say, “TFs+500” (resp. “TFs+1000”) represents the gene set including highly variable TFs and top 500 (resp. 1000) most-varying genes. 28 benchmark datasets were then created by combining the 14 subdatasets with two kinds of “ground truth” regulatory relationships: the functional interaction network String [27] and a non-specific ChIP-Seq network [28]. Detailed information regarding the eight datasets is presented in Table 1, where column “Cells” (resp. “Genes”) denotes cell (resp. gene) number in the expression data, column “TFs+500” (resp. “TFs+1000”) denotes the number of genes whose regulatory relationships were constructed based on regulatory networks, and column “GEO” represents Gene Expression Omnibus accession number.

To evaluate the performance of cell type embedding representation, four scRNA-seq datasets were applied: the Zeisel dataset [29], the peripheral blood mononuclear 4k cells from the 10X genomics platform (10X PBMC) dataset [30], the mouse bladder cells dataset [31], and the worm neuron cells dataset [32]. Genes expressed in less than 1% of cells, and cells contained fewer than 10 expressed genes were excluded from each dataset [19,33]. All cells of the datasets were annotated with known categories. Detailed information about the processed datasets is displayed in Table 2.

In addition, eight of eleven cell types in the human peripheral blood mononuclear 68k cells (68K PBMC) datasets [30], i.e., the types having more than 2000 cells, were used to train and evaluate the SIGRN model for generating scRNA-seq data. The raw datasets consisted of 68,579 cells and 32,738 genes. As conducted above, genes expressed in less than 1% of cells, and cells with fewer than 10 expressed genes were firstly removed from each dataset. Then each cell was normalized with the ‘normalize_per_cell’ function, so as to make its total expression value on all genes up to 20,000. Next, we chosen the top 1000 highly variable genes from the log-transformed normalized data and further standardized gene expression using the ‘normalize_per_cell’ function from Scanpy package 1.9.1 [34]. For each type of data, approximately 80% cells were chosen as the training set, and the rest were allocated to the test set. The specific number of cells for each type is provided in Table 3, where columns “Train" and “Test" refer to the number of cells used for training and testing, respectively.

All gene expression data were subjected to log2 transformation and Z-score normalization before they are fed into the deep learning methods.

### 2.2. Evaluation Metrics and Parameter Settings

Three kinds of evaluation metrics were adopted to estimate the performance of method SIGRN, and compared it with that of some leading-edge methods.

(1) GRN evaluation metrics

As detailed in refs. [16,19], the performance of GRN inference was assessed using the Early Precision Ratio (EPR) and the Area Under the Receiver Operating Characteristic Curve (AUC). For a given regenerated network, Early Precision is defined as “the proportion of true positives among the top-*k* edges” (*k* counts the edges in the “ground truth” network). EPR equals to the ratio of the measured Early Precision against the performance of a random predictor [35]. In the experiments, BEELINE [25] was called to calculate EPR and AUC.

Given the predicted weighted adjacency matrix *A*∈$R^{\left|G|\times |G\right|}$ and a threshold *t* (0 ≤ *t* ≤ 1), a binary adjacency matrix $A^{p}$ can be produced, where each entry $a_{ij}^{p}$ (*i*, *j* = 1, 2, …, |G|) is ascertained in terms of Formula (1):

(1) $$a_{ij}^{p}=\left\{\begin{array}{cc} 1, & \mathrm{if}\;a_{ij}>0,\\ 0, & \mathrm{otherwise}. \end{array}\right.$$

Let $A^{t}$ denote the |G| × |G| binary adjacency matrix of the ground truth GRN. True Positive (TP), True Negative (TN), False Positive (FP), and False Negative (FN) equal the number of 1 in matrices $A^{p}$⊙$A^{t}$, ∼$A^{p}$⊙∼$A^{t}$, $A^{p}$⊙∼$A^{t}$ and ∼$A^{p}$⊙$A^{t}$ (⊙ denotes the Hadamard product), respectively. The operator ∼ flips each entry of a matrix, i.e., changing 0 to 1 and vice versa. The Receiver Operating Characteristic (ROC) Curve can be plotted using True Positive Rate (TPR) and False Positive Rate (FPR), which are calculated as in Equations (2) and (3):

(2) $$TPR=\frac{TP}{TP+FN},$$

(3) $$FPR=\frac{TP}{FP+TN}.$$

(2) Cell representation evaluation metrics

We firstly clustered the cells into a given number of clusters in terms of their embedded representations with algorithm Louvain [36]. Then two prevalent clustering evaluation metrics, i.e., Adjusted Rand Index (ARI) and Normalized Mutual Information (NMI) [33], were adopted to estimate the effect of cell representation indirectly.

Given a group of |C| cells, let $P_{1}$ = {$P_{11}$,$P_{12}$,…,$P_{1k}$} and $P_{2}$ = {$P_{21}$,$P_{22}$,…,$P_{2k}$} denote the genuine and predicted partitions of cells into *k* clusters, respectively. The calculation of ARI is as Equation (4):

(4) $$ARI\left(P_{1},P_{2}\right)=\frac{2(ad-bc)}{\left(a+b)(b+d)+(a+c)(c+d\right)},$$

where *a* represents the number of pairs of cells that are in the same subset in $P_{1}$ and $P_{2}$. *b* denotes the number of pairs of cells that are in the same subset in $P_{1}$ but in different subsets in $P_{2}$. *c* equals the number of pairs of cells that are in different subsets in $P_{1}$ but in same subset in $P_{2}$. *d* denotes the number of pairs of cells that are in different subsets in $P_{1}$ and $P_{2}$. NMI is calculated as in Equation (5):

(5) $$NMI\left(P_{1},P_{2}\right)=\frac{2MI(P_{1},P_{2})}{H\left(P_{1}\right)+H\left(P_{2}\right)},$$

(6) $$MI\left(P_{1},P_{2}\right)=\sum_{i=1}^{k}\sum_{j=1}^{k}p\left(i,j\right)\log\frac{p(i,j)}{p_{1}\left(i\right)p_{2}\left(j\right)},$$

(7) $$H\left(P_{i}\right)=-\sum_{j=1}^{k}p_{i}\left(j\right)\log p_{i}\left(j\right),$$

where MI($P_{1}$,$P_{2}$) represents mutual information of $P_{1}$ and $P_{2}$, *H*($P_{i}$) (*i* = 1,2) represents the entropy of $P_{i}$, $p_{i}$(*j*) = $\frac{|P_{ij}|}{\left|C\right|}$, and *p*(*i*,*j*) = $\frac{|P_{1i}\cap P_{2j}|}{\left|C\right|}$.

(3) Data generation evaluation metrics

As performed in refs. [19,37,38], random forest (RF) [39] classifier was applied to classify genuine cells versus generated ones. A poorer classification performance indicates better generation quality. The AUC score was calculated to evaluate the classification effectiveness. Here True Positive (TP) (resp. False Negative (FN)) denotes the number of genuine cells which are correctly (resp. incorrectly) classified as the real (resp. generated) ones. True Negative (TN) (resp. False Positive (FP)) denotes the number of generated cells which are correctly (resp. incorrectly) classified as generated (resp. real) ones. In the experiments, five-fold cross-validation was employed to estimate the generalization capability of each classifier.

**Figure A1:**
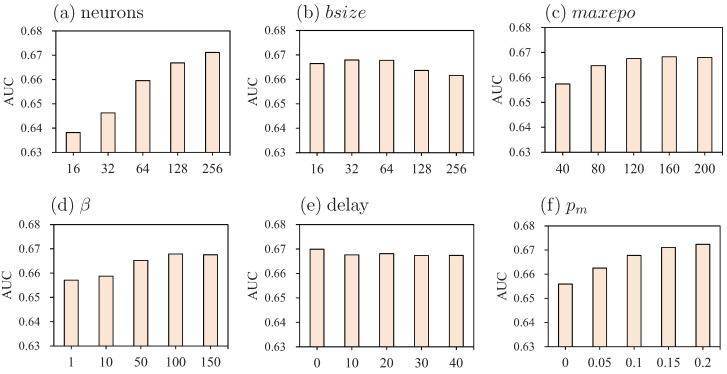
Comparisons of the AUC values under different parameter settings. Each result is the average of AUCs across the seven BEELINE datasets (“TFs+500”, String). For testing a certain parameter, the other parameters were set as follows, they are the same in the following figure: (**a**) $p_{m}$ = 0.1, bsize = 64, β = 100, maxepo = 120, “delay” was set to 30; (**b**) $p_{m}$ = 0.1, β = 100, maxepo = 120, “delay” was set to 30, “neurons” was set to 128; (**c**) $p_{m}$ = 0.1, bsize = 64, β = 100, “delay” was set to 30, “neurons” was set to 128; (**d**) $p_{m}$ = 0.1, bsize = 64, maxepo = 120, “delay” was set to 30, “neurons" was set to 128; (**e**) $p_{m}$ = 0.1, bsize = 64, β = 100, maxepo = 120, “neurons” was set to 128; (**f**) bsize = 64, β = 100, maxepo = 120, “delay” was set to 30, “neurons” was set to 128.

**Figure A2:**
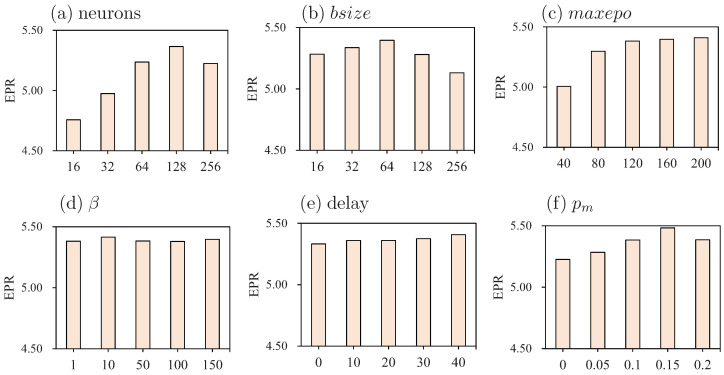
Comparisons of the EPR values under different parameter settings. Each result is the average of EPRs across the seven BEELINE datasets (“TFs+500”, String).

During the experiments, the mask augmentation probability $p_{m}$ was set to 0.1. The Adam optimiser was adopted to train the proposed SIGRN model. Both the encoder and decoder were implemented as three-layer multilayer perceptron (MLP) neural networks [19], having a hidden layer with 128 neurons and a tanh activation function. The learning rate was set to 1 $\times \;10^{-4}$ for the MLP network, and 2 $\times \;10^{-5}$ for the adjacency matrix *A* [19]. The other hyperparameters were configured as follows: bsize = 64, β = 100, and maxepo = 120. The L1 norm regularization in Equations (14) and (15) was introduced with a delay of 30 iterations. The values of these parameters were ascertained via abundant experimental tests, as shown in Figure A1 and Figure A2. The parameters for methods GRN-VAE and DeepSEM were set as in the corresponding literature. Each reported results represents the average across 10 runs.

### 2.3. Comparisons of GRN Inference Among Different Models

**Figure 1:**
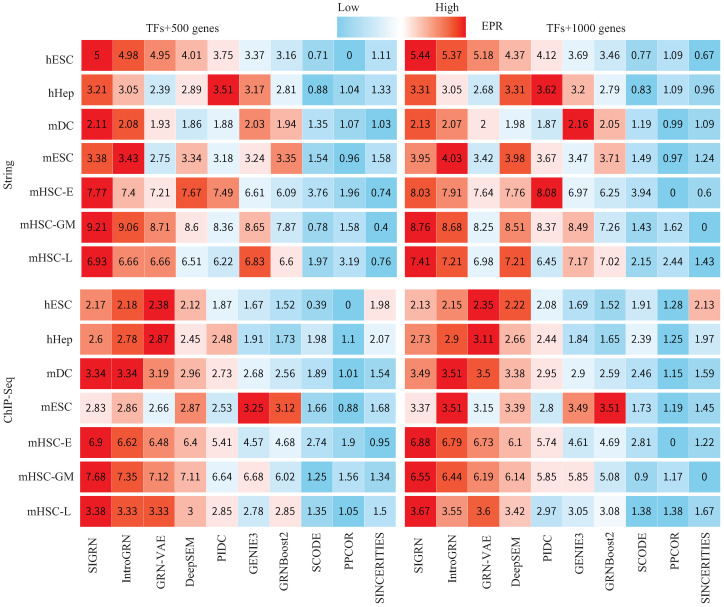
Comparisons of the EPR values: (1) “TFs+500”, String. (2) “TFs+1000”, String. (3) “TFs+500”, ChIP-Seq. (4) “TFs+1000”, ChIP-Seq. The color gradient spans from blue (low EPR values) to red (high EPR values).

In Figure 1, we compare the EPR values of method SIGRN against nine cutting-edge approaches using seven labeled BEELINE datasets. The comparison approaches include three deep learning methods IntroGRN [24], GRN-VAE [21] and DeepSEM [19], as well as six non-deep learning models: PIDC [4], GENIE3 [6], GRNBoost2 [7], SCODE [8], PPCOR [5], SINCERITIES [9]. Except for methods IntroGRN, GRN-VAE and DeepSEM, the EPR values of the other six methods were directly taken from ref. [19]. From Figure 1 we can notice that the deep learning methods generally perform better, with the SIGRN method acquiring the highest EPR in most instances. Additionally, for each dataset, significant tests were conducted between the SIGRN method and other deep learning methods, i.e., *t*-tests were applied on the EPR values of ten repeated experiments. The experimental results are show in Table A1, where column $p^{I}$ (resp. $p^{G}$ and $p^{D}$) indicates the *p*-values between methods SIGRN and IntroGRN (resp. GRN-VAE and DeepSEM). A *p*-value of less than 0.05 was considered significant. From the table, it is evident that method SIGRN achieves significantly superior EPR scores to methods IntroGRN, GRN-VAE, and DeepSEM in most cases.

In Table 4, the deep learning methods are further compared in terms of the AUC values (the significant experimental results are shown in Table A2). The results indicate that method SIGRN consistently achieves significantly higher AUC scores than methods GRN-VAE and DeepSEM in most cases. Although methods SIGRN and IntroGRN do not exhibit statistically significant difference in terms of AUC values, method SIGRN adopts fewer parameters compared with method IntroGRN.

**Figure 2:**
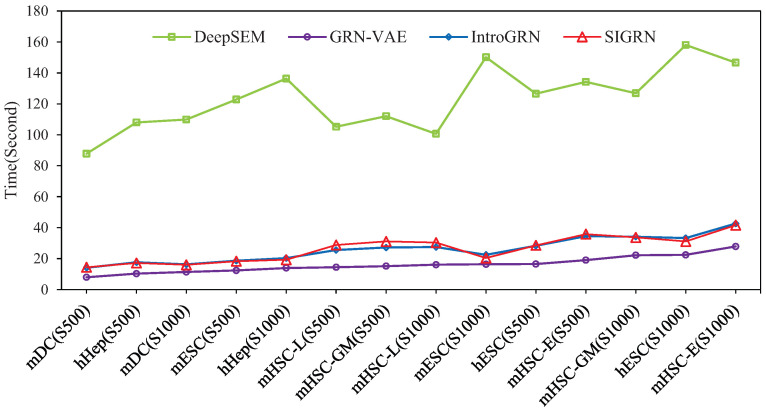
Comparisons of executing time among the four deep learning methods.

In Figure 2, the running time of the four deep learning methods is compared. As illustrated in this figure, methods SIGRN and IntroGRN present a similar efficiency. They cost longer training time than method GRN-VAE due to their adversarial training, and execute much faster than the DeepSEM method.

Furthermore, in order to evaluate the performance of method SIGRN more comprehensively, comparisons were conducted with the recently proposed supervised learning method STGRNS [16]. In the experiments, instances of two scales ground-truth datasets were generated. In the small-size (resp. large-size) instances, 10 (resp. 90) percent of all edges were designated as the training set, and the remainder were adopted as the test set. In Table 5, column STGRNS-S (resp. STGRNS-L) represents the results of method STGRNS on small-size (resp. large-size) instances. From this table, it can be discovered that although the performance of the two methods is comparable for solving large-size instances, method SIGRN can exhibit obvious advantage when dealing with the small-size instances. The experimental results reflect that supervised algorithms may struggle to achieve good performance when labeled data are scarce, whereas unsupervised ones may offer greater flexibility.

**Figure 3:**
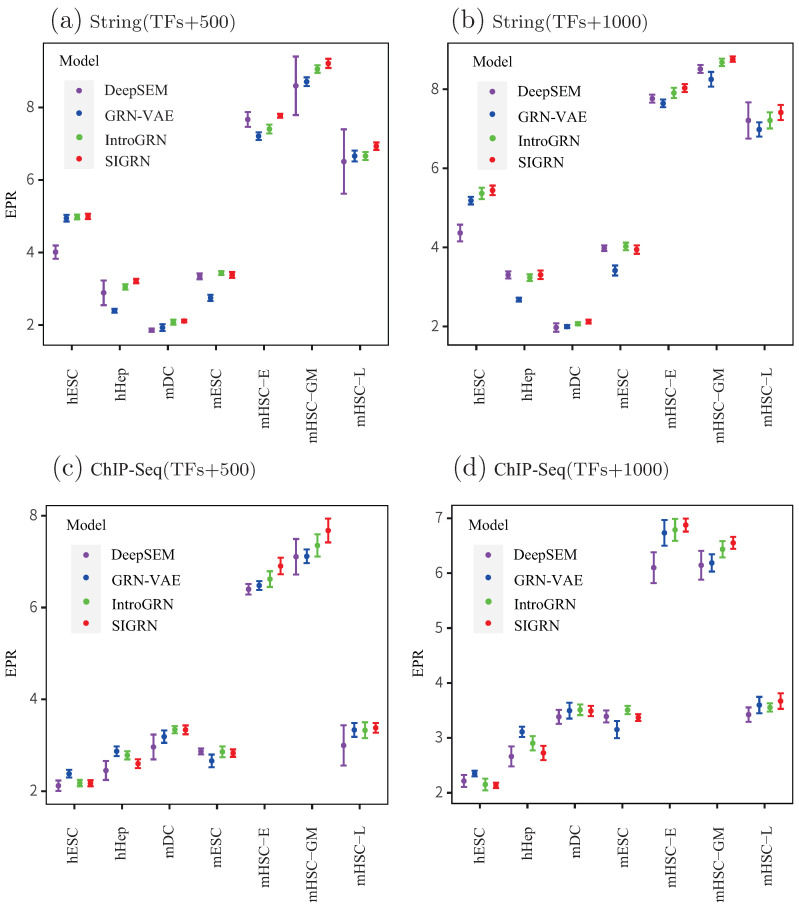
Stability comparisons among four deep learning methods. Error bars represent the standard deviation calculated from 10 experiments, with shorter bars representing greater stability.

Given that stability is crucial for neural network models, we evaluate it for the four neural network based methods. As illustrated in Figure 3, the horizontal and vertical axes denote datasets and the EPR, respectively. Error bars represent the standard deviation calculated from 10 experiments. It is evident that methods SIGRN, IntroGRN, and GRN-VAE exhibit greater stability than the DeepSEM method, as indicated by their relatively shorter error bars. The average standard deviations over the 28 benchmark datasets are as follows: 0.2316 for DeepSEM, 0.1186 for GRN-VAE, 0.1132 for IntroGRN, and 0.1024 for SIGRN. It is obvious that method SIGRN demonstrates higher stability compared to the other ones.

**Figure 4:**
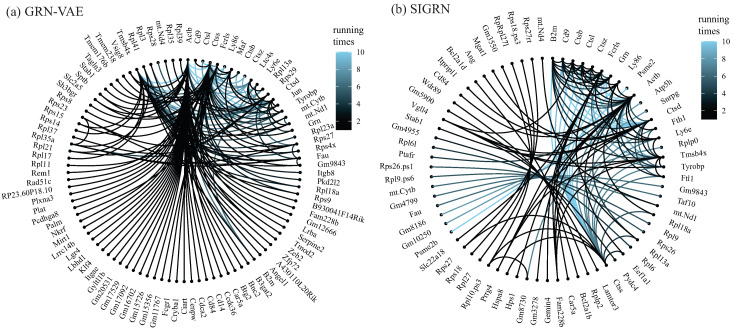
The frequency of the edges in *E*50 across the ten replicates. (**a**) method GRN-VAE. (**b**) method SIGRN. The color gradient spans from black (less times) to sky blue (more times).

We further assessed the stability of methods SIGRN and GRN-VAE on the large-scale unlabeled dataset Saline. As performed by Zhu et al. [21], 5000 highly variable genes, i.e., they have significantly different gene expression on different cells, were chosen with the R package Seurat 5.0.3. We repeated the experiments for ten times. For the *i*th experiment, let $A_{i}$ (*i* = 1, 2, …, 10) represent the produced adjacency matrix, and edge set $E100_{i}$ (resp. $E50_{i}$, $E25_{i}$, $E15_{i}$) records the top 100 (resp. 50, 25, 15) predicted edges with the largest absolute weights. Let E100 = ${⋃}_{i=1}^{10}$$E100_{i}$, E50 = ${⋃}_{i=1}^{10}$$E50_{i}$, E25 = ${⋃}_{i=1}^{10}$$E25_{i}$, and E15 = ${⋃}_{i=1}^{10}$$E15_{i}$. Their size is 299, 126, 53, and 33 for method SIGRN (|E100| = 299, |E50| = 126, |E25| = 53, |E15| = 33), and 352, 156, 69, and 47 for method GRN-VAE (|E100| = 352, |E50| = 156, |E25| = 69, |E15| = 47). Figure 4 visualizes the stability results of extracting top 50 predicted edges from each replicate, i.e., how often the edges in *E*50 appear across the ten replicates. From the figure we notice that method SIGRN exhibits stronger stability than method GRN-VAE. Fourteen edges are consistently repeated across the SIGRN replicates, 33 edges appears more than five times, and 37 edges are seen only once. Nevertheless, for method GRN-VAE, only eight edges are consistent repeated across the ten replicates, the edges that appears more than five times and only once are 32 and 81, respectively.

**Figure 5:**
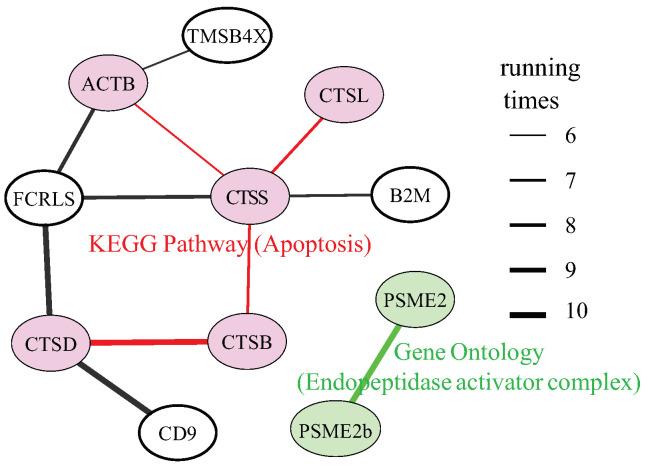
Top 15 regulatory relationships with the largest weights. The edge thickness indicates the number of times it is produced across ten replicate experiments. Genes with the same color are enriched in the same pathway.

In addition, among edge set E15 produced by method SIGRN, the edges (excluding the ones linking pseudogenes) that appear more than five times across the ten replicates are retained and exhibited, as shown in Figure 5. The edge thickness denotes the number of times it is produced across ten replicate experiments. As can be seen in this figure, genes marked with pink color (ACTB, CTSB, CTSL, CTSS, CTSD) are enriched in the Apoptosis Pathway, referred to the Kyoto Encyclopedia of Genes and Genomes (KEGG) database [40]. The Apoptosis Pathway is responsible for initiating, mediating, executing and regulating apoptosis, and plays essential role in developing and maintaining tissue homeostasis [41]. (TMSB4X, ACTB) [42] and (B2M, CTSS) [43] have been ascertained as two co-expression patterns. For example, Ling et al. [44] have reported genes TMSB4X and ACTB co-express in the E15.5 mouse cortical plate, and may play a collaborative role in early development of cortical cells. Genes PSME2 and PSME2b (marked in green) are a pair of co-expression ones, and they are annotated to endopeptidase activator activity according to the Gene Ontology (GO) database [45].

### 2.4. The Clustering Results from the Cell Representation

**Figure 6:**
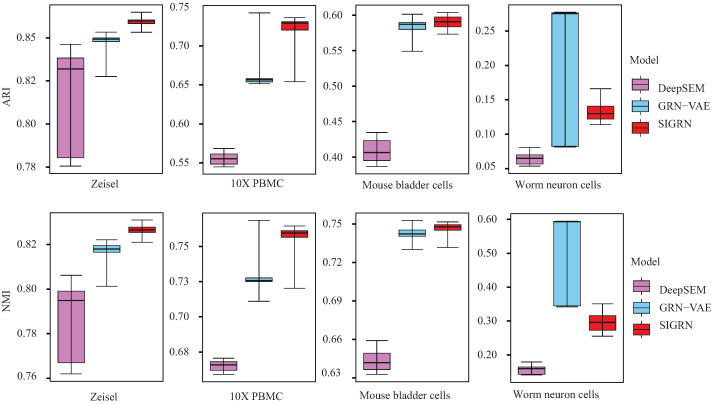
Box plots show clustering performance over four single-cell datasets.

**Figure 7:**
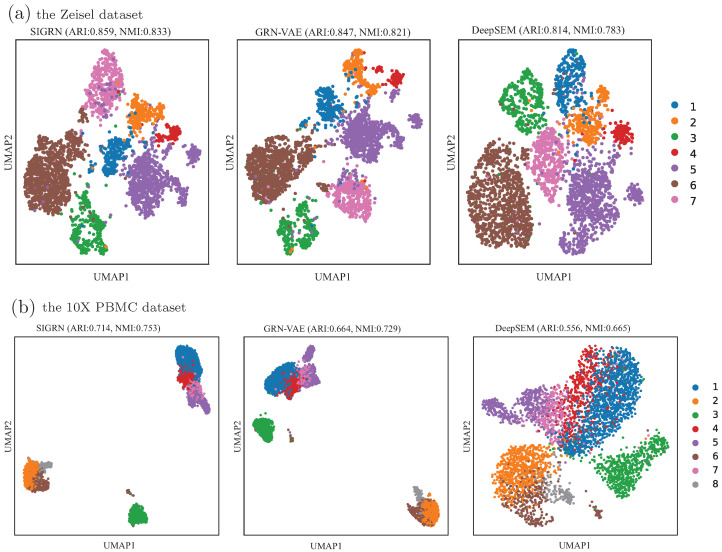

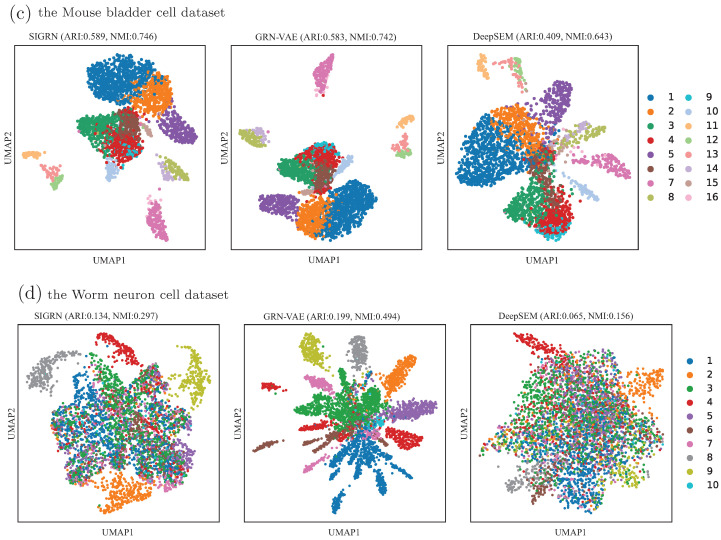
UMAP visualization of hidden representation. The ARI and NMI values are the average results from ten experiments, with higher values indicating better clustering performance.

As illustrated above, in the SIGRN method, an introspective adversarial training is executed from the perspective of cell representation in addition to cell generation. Hence the performance of learning embedded cell representation is evaluated and analysed. In the experiments, the top 1000 highly variable genes were selected for each dataset with scanpy package 1.9.1, and the corresponding zero proportions of datasets Zeisel, 10X PBMC, Mouse bladder cells, and Worm neuron cells were 0.315, 0.478, 0.585, and 0.896, respectively. In Figure 6, the performance of clustering cells by using their embedded representations are illustrated with box plots. Ten runs were executed for each dataset. From this figure we can notice that, the SIGRN method outperforms methods DeepSEM and GRN-VAE on Zeisel, 10X PBMC and Mouse bladder cells datasets regarding both ARI and NMI. It is worth noting that for dataset Worm neuron cells having a high proportion of zeros, both ARI and NMI of method DeepSEM are close to 0. Although method GRN-VAE can acquire higher ARI or NMI than method SIGRN, its performance stability seems weak. Furthermore, in order to depict the clustering results more intuitively, we visualized them through scatter plots. As exhibited in Figure 7, the clustering results of methods DeepSEM, GRN-VAE, and SIGRN on four datasets are depicted by applying Uniform Manifold Approximation and Projection (UMAP). From these figures it can be concluded that for most datasets, method SIGRN presents a better cell representation than methods GRN-VAE and DeepSEM, indicating the effectiveness of applying introspective adversarial in the training process.

### 2.5. Generation of scRNA-Seq Data

**Figure 8:**
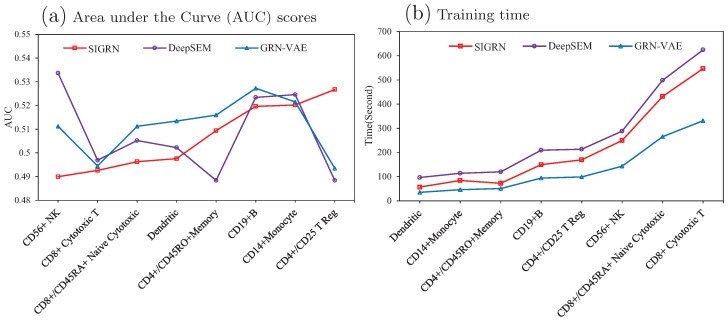
Comparisons of generating scRNA-seq data. (**a**) AUC scores from five-fold cross validation. (**b**) Executing time for training models.

The PBMC dataset was adopted to test and compare the performance of generating data among method GRN-VAE, DeepSEM, and SIGRN. Figure 8a demonstrates the generative performance of method SIGRN compared to methods GRN-VAE and DeepSEM. From this figure it can be seen that, except for datasets CD4+/CD45RO+Memory and CD4+/CD25 T Reg, method SIGRN acquires lower AUC than the other two ones. That is to say, the SIGRN method achieves superior performance on six out of eight datasets. In Figure 8b, the training time is compared among the three methods. The proposed method SIGRN has a longer running time than method GRN-VAE due to implementing adversarial training.

**Figure 9:**
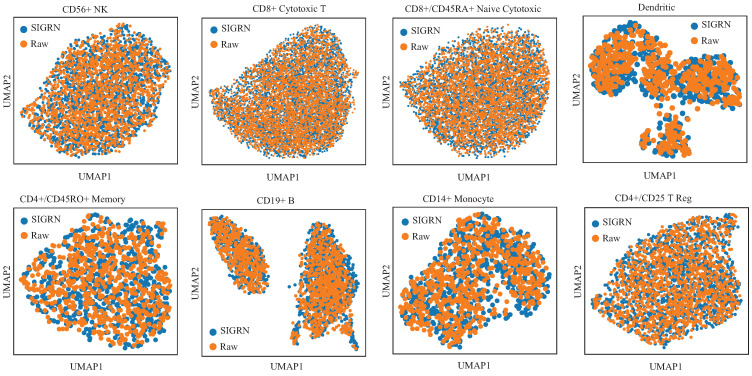
UMAP visualization of true data and generated data in SIGRN. The raw data is marked in orange color, and the data generated by method SIGRN is marked in blue color.

In Figure 9, the datasets generated with method SIGRN are visualized by using UMAP plots. Orange dots represent raw samples, while blue ones represent generated samples. The plots demonstrates that the scRNA-seq data generated by method SIGRN closely resemble the raw one, indicating that method SIGRN is capable of generating high-quality scRNA-seq data.

### 2.6. Model Interpretability and Ablation Experiments

**Figure 10:**
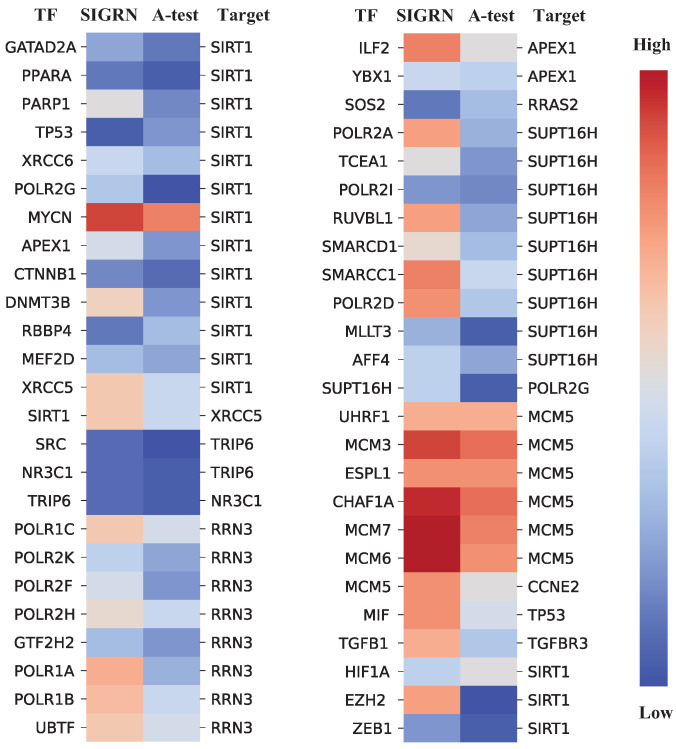
Comparison of gene correlations. Columns SIGRN and A-test indicate the correlations among genes based on the expression data generated by SIGRN and A-test, respectively.

To bolster the interpretability of the SIGRN model, the known regulatory relationships acquired from the String network were adopted to validate the SIGRN model on the hESC dataset. It is hypothesized that if there is a certain relationship between a transcription factor (TF) and its target gene, the expression values of them in the corresponding cells should exhibit a certain degree of correlation. Based on this hypothesis, fifty known regulatory relationships were chosen from the String network [27]. For each regulatory relationship, the genes of columns ‘Gene1’ and ‘Gene2’ [25] were specified as TFs and target genes, respectively. Let A-test denote the method without considering adjacency matrix *A*. We calculated the Spearman correlation coefficient for each pair of TF and target gene based on the expression data generated by method SIGRN, and those generated by method A-test. As illustrated in Figure 10, compared to method A-test, the pairs of TF and target present stronger correlation based on the expression data generated by method SIGRN, which indirectly supports the effectiveness of the SIGRN method in inferring GRN.

To assess the model effectiveness, a series of ablation experiments were conducted on seven BEELINE datasets (“TFs+500”, String). As shown in Table 6, the model performance is evaluated in terms of the AUC and the EPR values under the circumstances of adopting soft introspective adversarial mechanism or not, denoted as SIGRN and DSIGRN respectively. From this table we can see that, the SIGRN model outperforms the DSIGRN model on the AUC and the EPR values except for the mHSC-L dataset. The introduction of soft introspective adversarial mechanism does work.

In addition, as mentioned before, *L*1 norm regularization is used to prevent the inferred adjacency matrix *A* from getting too noisy, which contributes to improve the performance of GRN inference. Its effectiveness was also validated through ablation experiments. In Table 7, the model performance of adopting *L*1 norm regularization or not is evaluated. It can be seen from this table that when adopting *L*1 norm regularization, the EPR values are improved on four datasets, while the AUC values are increased on each of the datasets. Therefore, it also does work for *L*1 norm regularization to improve the model.

In Table 8, the model performance is compared between the two cases of applying mask augmentation ($p_{m}$ = 0.1) or not ($p_{m}$ = 0). It can been seen from this table that the adoption of mask augmentation indeed contributes to the model training, for the EPR values are increased on four datasets, and the AUC values are improved on each of the datasets.

As mentioned above, The L1 norm regularization is applied with a delay mechanism, whose effectiveness is demonstrated in Table 9. The experimental results still indicate that the delay mechanism does facilitate the model training.

## 3. Methods

**Figure 11:**
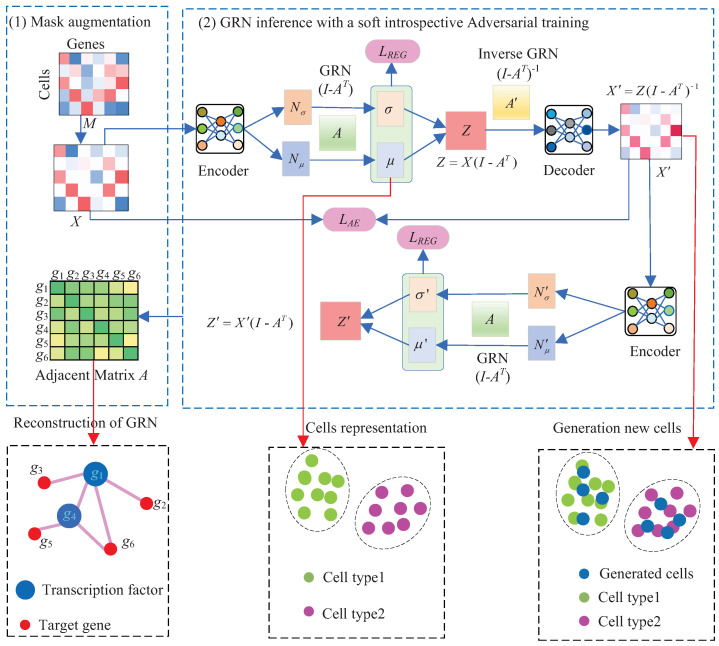
The overview of method SIGRN. It consists of two parts: (1) mask augmentation. (2) GRN inference with a soft introspective adversarial training. The decoder and encoder of a VAE is also employed as a pair of generator and discriminator.

Suppose that *X* is a |C| × |G| single-cell gene expression dataset, recording the expression levels of gene set *G* in a group of cells *C*. The GRN inference problem is defined as identifying the regulatory relationships among the |G| genes. That is to say, it aims to infer a weighted adjacency matrix *A*∈$R^{\left|G|\times |G\right|}$ from the matrix *X*. In this paper, we devise a soft introspective adversarial deep generative model SIGRN by generalizing the linear structural equation modeling framework and introducing adversarial mechanism in building a VAE model. The SIGRN method consists of two parts: mask augmentation and GRN inference with a soft introspective adversarial training, as exhibited in Figure 11.

### 3.1. Mask Augmentation

**Figure 12:**
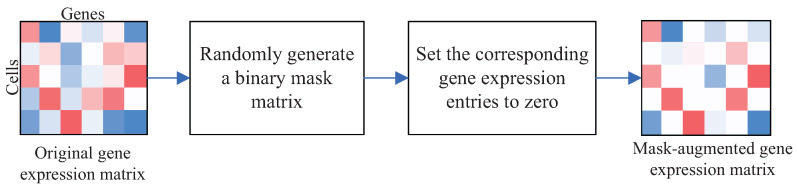
The block schema of the mask augmentation algorithm.

Research has indicated that the frequent occurrence of zeros in scRNA-seq data can be attributed to either the genuine absence of expression or missing data [46]. To establish a robust model suitable for the distribution of zeros [21], we apply mask augmentation on the single-cell gene expression matrix *X*. Figure 12 illustrates the block schema of the mask augmentation algorithm. The algorithm is primarily carried out in the following two steps: (1) Randomly generate a binary mask matrix that has the same dimension as the original gene expression matrix *X*. (2) The entries of matrix *X* that correspond to zero entries in the mask matrix are set to zero. The mask-augmented matrix is also represented by *X*. A detailed description of this process is provided in Appendix A.1.

### 3.2. GRN Inference with a Soft Introspective Adversarial Training

SEM is a robust multivariate statistical method that investigates the correlations between observed and unobserved (hidden) variables [47]. In this study, it is utilized to infer GRN by modeling the conditional dependencies among various genes. Let *A*∈$R^{\left|G|\times |G\right|}$ represent the weighted adjacency matrix of a GRN, it is assumed that a gene’s expression is ascertained with the weighted sum of the expressions of its regulators, as shown in Formula (8).

(8) $$X=XA^{T}+Z,$$

where *Z*∈$R^{\left|C|\times |G\right|}$ denotes a Gaussian noise matrix. The formula is further rearranged into Formulas (9) and (10):

(9) $$Z=X(I-A^{T}),$$

(10) $$X=Z{\left(I-A^{T}\right)}^{-1},$$

where *I*∈$R^{\left|G|\times |G\right|}$ is the identity matrix. The two formulas conform to a VAE framework naturally, with Formulas (9) and (10) respectively serving as the encoder and the decoder. The evidence lower bound (ELBO), as expressed in Formula (11), is then attempted to be maximized.

(11) $$ELBO=E_{Z∼q_{\phi }\left(Z|X\right)}\left[\log p_{\theta }\left(X|Z\right)\right]-D_{KL}\left(q_{\phi }\left(Z|X\right)∥p_{\theta }\left(Z\right)\right),$$

the two terms respectively denote the reconstruction loss and the Kullback-Leibler (KL) divergence. $q_{\phi }$(*Z*|*X*) and $p_{\theta }$(*X*|*Z*) represent a pair of encoder and decoder, ϕ and θ are model parameters.

As previously noted, the GRN-VAE method [21] improves the robustness of learning model by simulating dropouts with an additional classifier. In this study, an IAN is constructed by applying the decoder and encoder of a VAE as a pair of generator and discriminator, refraining from training additional neural networks. Let $L_{AE}$ and $L_{REG}$ denote the negative versions of the two terms in the ELBO.

(12) $$L_{AE}=-E_{Z∼q_{\phi }\left(Z|X\right)}\left[\log p_{\theta }\left(X|Z\right)\right],$$

(13) $$L_{REG}=D_{KL}\left(q_{\phi }\left(Z|X\right)∥p_{\theta }\left(Z\right)\right).$$

**Figure 13:**
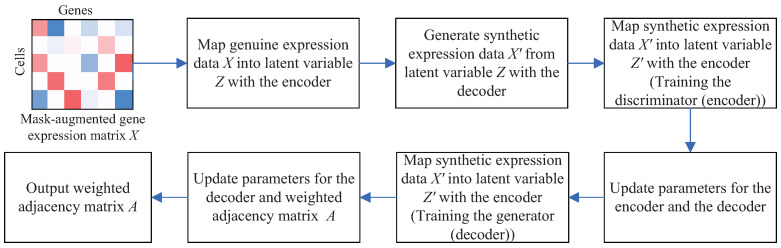
The block schema of the proposed SIGRN model.

$L_{AE}$ represents the expected negative reconstruction error, aiming to reduce the discrepancy between genuine and synthetic data. $L_{REG}$ contributes to the approximate posterior $q_{\phi }\left(Z|X\right)$ approaching the prior $p_{\theta }\left(Z\right)$, minimizing the discrepancy between the hidden variables encoded by real data and those sampled from the prior distribution. In addition, in the SIGRN model, $L_{REG}$ is also employed as the loss function of the IAN. As exhibited in Figure 11, the encoder, acting as a discriminator, tries to promote the posterior $q_{\phi }$(*Z*|$X^{\prime }$) to deviate from the prior $p_{\theta }$(*Z*) by maximizing $L_{REG}$, i.e., the discrepancy between the hidden variables encoded by generated data and those sampled from the prior distribution. Conversely, the decoder attempts to generate data $X^{\prime }$ that minimize $L_{REG}$, so that the posterior distribution $q_{\phi }$(*Z*|$X^{\prime }$) closely aligns with the prior distribution $p_{\theta }$(*Z*). The loss functions of the SIGRN model are designed as Formulas (14) and (15):

(14) $$L_{E}=E\left(X\right)+exp\left(-E\left(X^{\prime }\right)\right)+\beta ∥A∥+L_{AE},$$

(15) $$L_{D}=E\left(X^{\prime }\right)+\beta ∥A∥+L_{AE},$$

where *E*(*X*) = $D_{KL}$($q_{\phi }$(*Z*|*X*)∥$p_{\theta }$(*Z*)). Exponential function exp(·), performed as a soft threshold function to maximize $E(X^{\prime })$, is adopted to replace the hard threshold term $\alpha {\left[m-E\left(X^{\prime }\right)\right]}^{+}$ (${\left[\cdot \right]}^{+}$ = max(0, ·), *m* is a hard positive margin) in method IntroGRN [24]. As expressed in the hard threshold term, its value is set to 0 when $E(X^{\prime })$ ≥ *m*, leading to the disappearance of gradient of the IntroGRN discriminator network. This may play a negative role in the stability of training. The introduction of soft threshold function eliminates the use of additional hyperparameters *m* and α, exempting the term from being restricted by the margin and avoiding gradient disappearance effectively. It not only makes the model easier to optimize but also enhances the stability of model training. A smaller exp($E(X^{\prime })$) contributes to a larger $E(X^{\prime })$. ∥A∥ denotes L1 norm regularization, which aids in constraining the parameters of matrix *A* and keeps it from becoming too noisy. $L_{AE}$ represents the reconstruction error. The block schema of training the SIGRN model is described in Figure 13. The training is implemented primarily by the following six steps: (1) Map genuine expression data *X* into latent variable *Z* with the encoder. (2) Generate synthetic expression data $X^{\prime }$ from latent variable *Z* with the decoder. (3) Map synthetic expression data $X^{\prime }$ into latent variable $Z^{\prime }$ with the encoder (training the discriminator to identify synthetic expression data as fake). (4) Update parameters for the encoder and the decoder. (5) Map the synthetic expression data $X^{\prime }$ into latent variable $Z^{\prime }$ with the encoder (training the generator to produce real-like expression data). (6) Update the parameters for the decoder and the weighted adjacency matrix *A*. Algorithm 1 further depicts the process in detail.
**Algorithm 1** Training SIGRN model**Input:** A |C| × |G| single-cell gene expression data *X*,the number of epochs maxepo, multilayer perceptron learning rate $\eta_{1}$, adjacency matrix learning rate $\eta_{2}$, batch size bsize, parameter β **Output:** matrix $A\in R^{\left|G|\times |G\right|}$
  1:Initialize *A*, θ, and ϕ randomly  2:$X_{buf}$ = *X*  3:**for**epo←1 to maxepo**do**  4:    **for** *b*←1 to ⌈$\frac{\left|C\right|}{bsize}$⌉ **do**  5:        *X*←bsize random samples from $X_{buf}$  6:        $N_{\mu }$, $N_{\sigma }$←**Encoder**(*X*)  7:        μ←$N_{\mu }$(*I*−$A^{T})$, σ←$N_{\sigma }$(*I*−$A^{T})$  8:        *Z*←μ+σϵ (ϵ∼N(0, *I*))  9:        $X^{\prime }$←**Decoder**(*Z*($I-A^{T}{)}^{-1}$)10:        $L_{AE}$←$E_{Z∼q_{\phi }\left(Z|X\right)}$[$\log p_{\theta }\left(X|Z\right)]$11:        $N_{\mu^{\prime }}$, $N_{\sigma^{\prime }}$←**Encoder**($X^{\prime }$)12:        $\mu^{\prime }$←$N_{\mu }^{\prime }\left(I-A^{T}\right)$, $\sigma^{\prime }$←$N_{\sigma }^{\prime }\left(I-A^{T}\right)$13:        $Z^{\prime }$←$\mu^{\prime }$+$\sigma^{\prime }$ϵ (ϵ∼N(0,I))14:        $L_{E}$←E(X)+$\exp(-E\left(X^{\prime }\right))$+β∥A∥+$L_{AE}$15:        ϕ, θ←$\eta_{1}$$\nabla_{\phi ,\theta ,A}$($L_{E})$                            ▹ Perform Adam updates for θ, ϕ16:        $N_{\mu^{\prime }}$, $N_{\sigma^{\prime }}$←**Encoder**($X^{\prime }$)17:        $\mu^{\prime }$←$N_{\mu }^{\prime }\left(I-A^{T}\right)$, $\sigma^{\prime }$←$N_{\sigma }^{\prime }\left(I-A^{T}\right)$18:        $Z^{\prime }$←$\mu^{\prime }$+$\sigma^{\prime }$ϵ (ϵ∼N(0, *I*))19:        $L_{D}$←*E*($X^{\prime }$)+β∥A∥+$L_{AE}$20:        θ, *A*←$\eta_{1}$$\eta_{2}$$\nabla_{\theta ,A}$($L_{D}$)                            ▹ Perform Adam updates for θ, *A*21:    **end for**22:**end for**


As illustrated in Algorithm 1, the algorithm begins with randomly initializing the adjacency matrix *A*, as well as parameters θ and ϕ associated with the model. As exhibited in Figure 11, the mask augmented matrix *X* is fed into the encoder, producing two matrix variables $N_{\mu }$ and $N_{\sigma }$ with the same dimensions as *X*. Then the latent variable *Z* is obtained from a pair of matrix variables μ and σ, which are generated by integrating the relationships between genes into variables $N_{\mu }$ and $N_{\sigma }$, respectively. As illustrated above, the encoder and decoder are respectively used as discriminator and generator in constructing an introspective adversarial training. Therefore, in steps 11 to 13 (resp. steps 16–18), the generated matrix $X^{\prime }$ is also input into the encoder. The terms $\exp(-E\left(X^{\prime }\right))$ in step 13 and *E*($X^{\prime }$) in step 19 represent the loss of discriminator and generator, respectively. Particularly, for evaluating the quality of data generated by the decoder, the errors *E*($X^{\prime }$) in step 19 are back-propagated to the decoder, updating the decoder’s parameter θ, as shown in step 20.

## 4. Discussion

**Figure 14:**
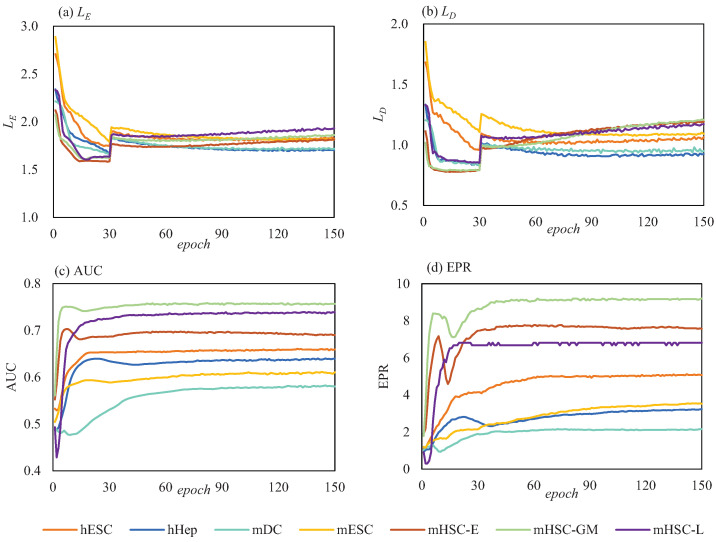
Losses and performance of the SIGRN model with epoch steps.

In this study, aiming at the shortcoming of VAE generating poor-quality data, we introduce a GRN inference framework by introducing adversarial mechanism in constructing a VAE model. It applies “soft" introspective adversarial mode to avoid training additional neural networks and adding additional training parameters. Since the performance of the SIGRN model is susceptible to the pre-existing features of raw scRNA-seq data, e.g., the diversity of gene expression distribution, and the prevalence of zero values, a series of preprocessing operations such as log2 transformation, Z-score normalization, as well as mask augmentation were implemented to reduce the difficulty of model fitting and increase the speed of convergence. Furthermore, the selection of network depth and hyperparameters is also a critical factor that affects model learning, for inadequate settings can result in gradient explosion or gradient vanishing. Hence extensive preliminary experiments have been conducted to explore the optimal settings. In Figure 14, seven BEELINE datasets (“TFs+500”, String) were applied to test the loss function values at each epoch. From Figure 14a,b we can see that both $L_{E}$ and $L_{D}$ drastically decrease at first and then gradually flatten out. Particularly, due to the introduction of L1 norm regularization with a delay of 30 iterations, there is a sudden change of $L_{E}$ and $L_{D}$ at epoch 30. Furthermore, both EPR and AUC gradually reach a relatively stable value with the increase of epoch, as exhibited in Figure 14c,d. The faster convergence suggests that the SIGRN model can reach a relatively better performance in less time.

During the experiments, it is discovered that the model is still constrained by multiple parameters and unable to produce high-quality data in the presence of a large number of zero values. Better generative models, integrating more types of single cell omics data such as scATAC-seq and DNA methylation, should be explored to enhance gene network inference. By incorporating protein-protein interaction network into the generative model along with scRNA-seq, scATAC-seq, and DNA methylation data, the attention mechanism may be applied to focus on the most relevant interactions to improve the accuracy of gene network inference. It is also noticed that the training time increases rapidly with the increase in size of datasets. For example, the running time is 56.8 s for the smallest dataset “Dendritic”, and increases almost ten times for the largest one “CD8+ Cytotoxic T”. Therefore, it is critical to filtering the genes with less information so as to improve the overall training efficiency. That is to say, it is recommended to preselect some feature genes, e.g., highly variable genes, to reduce the scale of training samples. A multi-core parallel training mode may also be applied to increase the training efficiency. In addition, the SIGRN model needs to be further improved in terms of interpretability. The causal inference method, which can elucidate the regulatory relationships among genes, will be studied in future work to improve the model interpretability.

## 5. Conclusions

It is a significant challenge in bioinformatics to extracting potential gene regulatory relationships from scRNA-seq data using computational techniques. In recent years, a number of unsupervised deep learning methods have been put forward to solve the problem by using VAE and SEM. Owing to the shortcoming of VAE generating poor-quality data, this study introduces a novel inference model SIGRN which incorporates an adversarial mechanism into the VAE framework. In this model, an IAN is created by respectively adopting the decoder and encoder of VAE as generator and discriminator. A new loss function is devised by minimizing the difference between real data and generated data, the hidden variables encoded by real data and the hidden variables sampled from the prior distribution, as well as maximizing the hidden variables encoded by generated data and the hidden variables sampled from the prior distribution. Moreover, the introduction of “soft" threshold function diminishes the number of training parameters. Experimental results have demonstrated that the GRNs inferred using method SIGRN exceed those derived from various cutting-edge methods across the majority of benchmark datasets. SIGRN still achieves better performance on representing cells and generating scRNA-seq data in most datasets. Therefore, method SIGRN may become an effective supplementary tool for inferring GRNs.

## Figures and Tables

**Table 1:** Datasets for evaluating the performance of inferring GRN. The symbol “- -” indicates the absence of known “ground truth” regulatory relationships.

Dataset	Cells	Genes	TFs+500	TFs+1000	GEO
hESC	758	17,735	910	1410	GSE75748
hHep	425	11,515	948	1448	GSE81252
mDC	383	7371	821	1321	GSE48968
mESC	421	18,385	1120	1620	GSE98664
mHSC-E	1071	4762	704	1204	GSE81682
mHSC-GM	889	4762	632	1132	GSE81682
mHSC-L	847	4762	560	692	GSE81682
Saline	2623	14,350	- -	- -	GSE121654

**Table 2:** Datasets for evaluating the performance of cell representation.

Dataset	Cells	Genes	Cell Types	Source
Zeisel mouse brain	3005	14,538	7	[29]
10X PBMC	4271	16,449	8	[30]
Mouse bladder cells	2746	19,079	16	[31]
Worm neuron cells	4186	11,955	10	[32]

**Table 3:** Datasets for evaluating the performance of data generation.

Annotated Cell Type	Cells	Train	Test	Source
CD8+ Cytotoxic T	20,773	16,673	4100	[30]
CD8+CD45RA+ Naive Cytotoxic	16,666	13,321	3345	
CD56+NK	8776	7079	1697	
CD4+/CD25 T Reg	6187	4903	1284	
CD19+B	5908	4677	1231	
CD4+/CD45RO+Memory	3061	2480	581	
CD14+Monocyte	2862	2293	569	
Dendritic	2099	1674	425	

**Table 4:** Comparisons of the AUC values among four deep learning methods. The best result for each dataset is shown in bold. It is the same in the following tables. The bold number refers to the best performer.

Dataset	TFs+500 Genes, String					TFs+1000 Genes, String			
	SIGRN	IntroGRN	GRN-VAE	DeepSEM		SIGRN	IntroGRN	GRN-VAE	DeepSEM
hESC	**0.6592**	0.6574	0.6551	0.6126		**0.6581**	**0.6579**	0.6546	0.6090
hHep	**0.6415**	0.6412	0.6303	0.6149		0.6204	**0.6216**	0.6094	0.5983
mDC	**0.5806**	0.5789	0.5546	0.5602		0.5644	**0.5650**	0.5408	0.5647
mESC	**0.6086**	0.6011	0.5954	0.5807		**0.6117**	0.6044	0.6006	0.5944
mHSC-E	0.6948	**0.7023**	0.6939	0.6644		0.6720	**0.6838**	0.6745	0.6500
mHSC-GM	0.7567	**0.7604**	0.7560	0.7337		0.7200	**0.7256**	0.7091	0.6915
mHSC-L	**0.7363**	0.7308	0.7261	0.6811		**0.7216**	0.7182	0.7082	0.6663
Dataset	TFs+500 genes, ChIP-Seq					TFs+1000 genes, ChIP-Seq			
	SIGRN	IntroGRN	GRN-VAE	DeepSEM		SIGRN	IntroGRN	GRN-VAE	DeepSEM
hESC	0.5335	0.5344	**0.5640**	0.5301		0.5388	0.5372	**0.5611**	0.5330
hHep	0.5445	0.5397	**0.5627**	0.5381		0.5465	0.5446	**0.5622**	0.5505
mDC	**0.5734**	0.5725	0.5643	0.5474		0.5615	**0.5623**	0.5538	0.5545
mESC	**0.5695**	0.5669	0.5694	0.5544		0.5703	0.5668	**0.5746**	0.5567
mHSC-E	**0.6339**	0.6318	0.6180	0.5905		**0.6199**	0.6168	0.5992	0.5774
mHSC-GM	**0.6770**	0.6744	0.6727	0.6379		0.6308	**0.6364**	0.6157	0.6005
mHSC-L	**0.6365**	0.6363	0.6358	0.5837		**0.6345**	0.6299	0.6300	0.5948

**Table 5:** Comparisons of the AUC values between the SIGRN and STGRNS methods. Columns STGRNS-S and STGRNS-L represent the results of the STGRNS method on small-size and large-size instances, respectively. The bold number refers to the best performer.

Dataset	TFs+500 Genes, String				TFs+1000 Genes, String		
	SIGRN	STGRNS-S	STGRNS-L		SIGRN	STGRNS-S	STGRNS-L
hESC	**0.659**	0.562	0.641		**0.658**	0.538	0.630
hHep	**0.642**	0.531	0.632		0.620	0.556	**0.630**
mDC	**0.581**	0.508	0.537		**0.564**	0.475	0.562
mESC	**0.609**	0.568	0.549		**0.612**	0.581	0.552
mHSC-E	**0.695**	0.516	0.672		0.672	0.576	**0.702**
mHSC-GM	**0.757**	0.427	0.633		0.720	0.523	**0.738**
mHSC-L	**0.736**	0.394	0.570		**0.722**	0.382	0.504
**Average**	**0.668**	0.501	0.605		**0.653**	0.519	0.617
Dataset	TFs+500 genes, ChIP-Seq				TFs+1000 genes, ChIP-Seq		
	SIGRN	STGRNS-S	STGRNS-L		SIGRN	STGRNS-S	STGRNS-L
hESC	0.533	0.522	**0.607**		0.539	0.513	**0.598**
hHep	0.545	0.541	**0.633**		0.547	0.572	**0.673**
mDC	0.573	0.513	**0.656**		0.562	0.507	**0.701**
mESC	0.570	0.549	**0.627**		0.570	0.565	**0.623**
mHSC-E	**0.634**	0.539	0.592		0.620	0.589	**0.624**
mHSC-GM	**0.677**	0.630	0.533		**0.631**	0.578	0.588
mHSC-L	**0.636**	0.416	0.507		**0.634**	0.503	0.528
**Average**	**0.595**	0.530	0.594		0.586	0.547	**0.619**

**Table 6:** Comparisons of introducing soft introspective adversarial mechanism. The bold number refers to the best performer.

Dataset	AUC			EPR	
	SIGRN	DSIGRN		SIGRN	DSIGRN
mDC	**0.581**	0.575		**2.114**	2.070
hHep	**0.642**	0.631		**3.215**	3.025
mESC	**0.609**	0.601		**3.380**	3.304
hESC	**0.659**	0.643		**4.997**	4.938
mHSC-L	0.736	**0.740**		6.932	**7.129**
mHSC-E	**0.695**	0.685		**7.767**	7.633
mHSC-GM	**0.757**	0.754		**9.213**	8.926

**Table 7:** Comparisons of introducing *L*1 norm regularization. The bold number refers to the best performer.

Dataset	AUC			EPR	
	L1 Norm	No L1 Norm		L1 Norm	No L1 Norm
mDC	**0.581**	0.568		**2.114**	2.034
hHep	**0.642**	0.615		**3.215**	3.057
mESC	**0.609**	0.597		**3.380**	3.295
hESC	**0.659**	0.651		**4.997**	4.907
mHSC-L	**0.736**	0.733		6.932	**7.220**
mHSC-E	**0.695**	0.686		7.767	**7.951**
mHSC-GM	**0.757**	0.748		9.213	**9.250**

**Table 8:** Comparisons of introducing mask augmentation. The bold number refers to the best performer.

Dataset	AUC			EPR	
	Mask	No Mask		Mask	No Mask
mDC	**0.581**	0.578		**2.114**	2.071
hHep	**0.642**	0.621		**3.215**	2.923
mESC	**0.609**	0.586		3.380	**3.475**
hESC	**0.659**	0.639		**4.997**	4.149
mHSC-L	**0.736**	0.730		6.932	**7.069**
mHSC-E	**0.695**	0.685		7.767	**7.772**
mHSC-GM	**0.757**	0.752		**9.213**	9.122

**Table 9:** Comparisons of introducing a delay mechanism. The bold number refers to the best performer.

Dataset	AUC			EPR	
	Delay	No Delay		Delay	No Delay
mDC	0.581	0.581		**2.114**	2.086
hHep	0.642	**0.644**		**3.215**	3.234
mESC	**0.609**	0.608		3.380	**3.383**
hESC	**0.659**	0.658		**4.997**	5.006
mHSC-L	**0.736**	0.730		**6.932**	6.887
mHSC-E	0.695	**0.705**		**7.767**	7.559
mHSC-GM	0.757	**0.760**		**9.213**	9.169

## Data Availability

The source code of SIGRN and the datasets applied in this study are available at https://github.com/lryup/SIGRN (accessed on 25 November 2024).

## Appendix A

### Appendix A.1. Mask Augmentation

Given the |C| × |G| gene expression matrix *X*, a binary mask matrix $M_{\left|C|\times |G\right|}$ that has the exact same size is generated. Each entry $m_{ij}$ (*i* = 1, 2, …, |C|, *j*=1, 2, …, |G|) of matrix *M* is independently sampled from a Bernoulli distribution with probability $p_{m}$, i.e., $m_{ij}$∼Bernoulli($p_{m}$). The mask-augmented matrix is also represented by *X*, where $x_{ij}$ = $x_{ij}$×$m_{ij}$ (*i* = 1, 2, …, |C|, *j* = 1, 2, …, |G|).

### Appendix A.2. Experimental Results Under Different Parameter Settings

Figure A1 and Figure A2 display the average AUC and EPR values for solving seven BEELINE datasets (“TFs+500”, String) under different parameter settings, i.e., the average results across the seven datasets were calculated and presented. In the figures, “neurons” means the number of neurons in a hidden layer, and “delay” means the iterations of delay when introducing L1 norm regularization. According to the acquired AUC and EPR values, the following values were chosen for the trade-off between performance and execution time, i.e., $p_{m}$ = 0.1, bsize = 64, β = 100, maxepo = 120, the number of neurons in a hidden layer was set to 128, and the iterations of delay was set to 30, which were also adopted in ref. [19].

### Appendix A.3. Significance Analysis of Experimental Results

For each dataset, the deep learning methods significance tests were conducted between the SIGRN method and the comparison methods, i.e., *t*-tests were applied on the EPR and AUC values of ten repeated experiments. As shown in Table A1 and Table A2, column $p^{I}$ (resp. $p^{G}$,$p^{D}$) indicates the *p*-values between methods SIGRN and IntroGRN (resp. GRN-VAE, DeepSEM). A *p*-value of less than 0.05 was considered significant. From the table, it is evident that method SIGRN still achieves significantly superior EPR scores to methods IntroGRN, GRN-VAE and DeepSEM in most cases. Although the difference between SIGRN and IntroGRN is not statistically significant in terms of AUC values, SIGRN maintains good performance while reducing the number of parameters compared to IntroGRN.

**Table A1:** Results of significant tests between SIGRN and other deep learning methods.

Dataset	TFs+500 Genes, String				TFs+1000 Genes, String		
	$p^{I}$	$p^{G}$	$p^{D}$		$p^{I}$	$p^{G}$	$p^{D}$
hESC	8.60 $\times \;10^{-27}$	7.79 $\times \;10^{-4}$	9.07 $\times \;10^{-15}$		6.00 $\times \;10^{-22}$	6.69 $\times \;10^{-7}$	1.87 $\times \;10^{-13}$
hHep	4.23 $\times \;10^{-20}$	8.83 $\times \;10^{-16}$	1.04 $\times \;10^{-7}$		2.36 $\times \;10^{-20}$	4.96 $\times \;10^{-14}$	5.75 $\times \;10^{-9}$
mDC	1.92 $\times \;10^{-13}$	5.01 $\times \;10^{-8}$	3.04 $\times \;10^{-3}$		1.37 $\times \;10^{-17}$	8.73 $\times \;10^{-12}$	0.479
mESC	4.93 $\times \;10^{-23}$	8.56 $\times \;10^{-15}$	1.82 $\times \;10^{-4}$		2.47 $\times \;10^{-21}$	3.83 $\times \;10^{-11}$	2.81 $\times \;10^{-5}$
mHSC-E	1.49 $\times \;10^{-16}$	7.12 $\times \;10^{-13}$	2.33 $\times \;10^{-2}$		5.96 $\times \;10^{-19}$	3.18 $\times \;10^{-11}$	6.91 $\times \;10^{-12}$
mHSC-GM	4.08 $\times \;10^{-19}$	5.85 $\times \;10^{-14}$	8.39 $\times \;10^{-3}$		1.40 $\times \;10^{-21}$	5.10 $\times \;10^{-15}$	6.84 $\times \;10^{-13}$
mHSC-L	5.90 $\times \;10^{-12}$	4.10 $\times \;10^{-5}$	3.18 $\times \;10^{-2}$		1.50 $\times \;10^{-3}$	6.57 $\times \;10^{-5}$	2.75 $\times \;10^{-2}$
Dataset	TFs+500 genes, ChIP-Seq				TFs+1000 genes, ChIP-Seq		
	$p^{I}$	$p^{G}$	$p^{D}$		$p^{I}$	$p^{G}$	$p^{D}$
hESC	1.72 $\times \;10^{-19}$	9.48 $\times \;10^{-15}$	0.376		4.46 $\times \;10^{-16}$	1.76 $\times \;10^{-12}$	0.388
hHep	1.63 $\times \;10^{-20}$	4.01 $\times \;10^{-9}$	1.67 $\times \;10^{-2}$		2.48 $\times \;10^{-18}$	1.92 $\times \;10^{-10}$	4.65 $\times \;10^{-2}$
mDC	1.22 $\times \;10^{-21}$	8.91 $\times \;10^{-5}$	8.69 $\times \;10^{-3}$		8.32 $\times \;10^{-21}$	0.150	0.514
mESC	1.03 $\times \;10^{-17}$	4.30 $\times \;10^{-10}$	3.00 $\times \;10^{-2}$		6.05 $\times \;10^{-24}$	4.50 $\times \;10^{-6}$	5.59 $\times \;10^{-5}$
mHSC-E	1.38 $\times \;10^{-20}$	2.06 $\times \;10^{-13}$	7.06 $\times \;10^{-11}$		4.22 $\times \;10^{-20}$	8.47 $\times \;10^{-8}$	1.21 $\times \;10^{-8}$
mHSC-GM	6.69 $\times \;10^{-18}$	2.08 $\times \;10^{-13}$	1.19 $\times \;10^{4}$		2.73 $\times \;10^{-21}$	2.53 $\times \;10^{-14}$	3.36 $\times \;10^{-7}$
mHSC-L	0.124	1.38 $\times \;10^{-11}$	6.57 $\times \;10^{-4}$		1.66 $\times \;10^{-4}$	1.13 $\times \;10^{-6}$	1.49 $\times \;10^{-2}$

**Table A2:** Results of significant tests between SIGRN and other deep learning methods.

Dataset	TFs+500 Genes, String				TFs+1000 Genes, String		
	$p^{I}$	$p^{G}$	$p^{D}$		$p^{I}$	$p^{G}$	$p^{D}$
hESC	1.56 $\times \;10^{-2}$	4.76 $\times \;10^{-4}$	8.27 $\times \;10^{-13}$		0.840	4.96 $\times \;10^{-4}$	1.07 $\times \;10^{-11}$
hHep	0.772	2.86 $\times \;10^{-7}$	1.91 $\times \;10^{-4}$		0.249	1.76 $\times \;10^{-4}$	1.17 $\times \;10^{-7}$
mDC	0.156	3.41 $\times \;10^{-13}$	1.19 $\times \;10^{-2}$		0.454	3.86 $\times \;10^{-6}$	0.943
mESC	1.13 $\times \;10^{-7}$	3.48 $\times \;10^{-9}$	2.80 $\times \;10^{-9}$		5.72 $\times \;10^{-6}$	2.82 $\times \;10^{-5}$	8.28 $\times \;10^{-10}$
mHSC-E	1.48 $\times \;10^{-4}$	0.627	1.59 $\times \;10^{-6}$		4.36 $\times \;10^{-7}$	0.257	4.28 $\times \;10^{-11}$
mHSC-GM	1.81 $\times \;10^{-3}$	0.541	4.08 $\times \;10^{-2}$		4.69 $\times \;10^{-3}$	7.90 $\times \;10^{-6}$	1.43 $\times \;10^{-11}$
mHSC-L	5.31 $\times \;10^{-3}$	3.94 $\times \;10^{-4}$	2.16 $\times \;10^{-3}$		0.133	4.21 $\times \;10^{-6}$	4.31 $\times \;10^{-4}$
Dataset	TFs+500 genes, ChIP-Seq				TFs+1000 genes, ChIP-Seq		
	$p^{I}$	$p^{G}$	$p^{D}$		$p^{I}$	$p^{G}$	$p^{D}$
hESC	0.444	1.77 $\times \;10^{-15}$	0.354		0.188	2.05 $\times \;10^{-11}$	2.04 $\times \;10^{-2}$
hHep	4.54 $\times \;10^{-3}$	4.79 $\times \;10^{-8}$	0.267		0.432	1.53 $\times \;10^{-6}$	0.226
mDC	0.639	3.20 $\times \;10^{-4}$	2.49 $\times \;10^{-2}$		0.735	3.88 $\times \;10^{-2}$	0.179
mESC	3.65 $\times \;10^{-2}$	0.879	2.64 $\times \;10^{-5}$		4.76 $\times \;10^{-3}$	2.44 $\times \;10^{-3}$	2.46 $\times \;10^{-6}$
mHSC-E	0.086	8.86 $\times \;10^{-9}$	6.89 $\times \;10^{-11}$		0.101	5.91 $\times \;10^{-12}$	7.17 $\times \;10^{-14}$
mHSC-GM	0.277	2.97 $\times \;10^{-2}$	3.25 $\times \;10^{-6}$		0.077	1.16 $\times \;10^{-5}$	1.83 $\times \;10^{-6}$
mHSC-L	0.924	0.706	2.64 $\times \;10^{-3}$		6.78 $\times \;10^{-3}$	3.81 $\times \;10^{-2}$	2.74 $\times \;10^{-3}$
